# Markerless *Escherichia coli rrn* Deletion Strains for Genetic Determination of Ribosomal Binding Sites

**DOI:** 10.1534/g3.115.022301

**Published:** 2015-10-04

**Authors:** Selwyn Quan, Ole Skovgaard, Robert E. McLaughlin, Ed T. Buurman, Catherine L. Squires

**Affiliations:** * Department of Biology, Stanford University, Stanford, California 094305; † Department of Science, Systems and Models, Roskilde University, DK-4000 Roskilde, Denmark; ‡ Department of Biosciences, Infection Innovative Medicines Unit, AstraZeneca R&D Boston, Waltham, Massachusetts 02451; § Department of Molecular Biology and Microbiology, Tufts University School of Medicine, Boston, Massachusetts 02111

**Keywords:** rrl, rrs, duplication, genomic instability, ribosome

## Abstract

Single-copy *rrn* strains facilitate genetic ribosomal studies in *Escherichia coli*. Consecutive markerless deletion of *rrn* operons resulted in slower growth upon inactivation of the fourth copy, which was reversed by supplying transfer RNA genes encoded in *rrn* operons *in trans*. Removal of the sixth, penultimate *rrn* copy led to a reduced growth rate due to limited *rrn* gene dosage. Whole-genome sequencing of variants of single-copy *rrn* strains revealed duplications of large stretches of genomic DNA. The combination of selective pressure, resulting from the decreased growth rate, and the six identical remaining scar sequences, facilitating homologous recombination events, presumably leads to elevated genomic instability.

The majority of antibiotic classes currently in clinical use act by inhibiting ribosome function. Their utility is threatened by the emergence of microbial resistance, mainly due to compound efflux (Thaker *et al.* 2010), covalent modification of the ribosome (Goossens 2009), or alteration of the antibiotic (Zhanel *et al.* 2012). The binding interactions of the antibiotics with the ribosome are established predominantly with its RNA components (Poehlsgaard and Douthwaite 2005), which in *Escherichia coli* are encoded by seven virtually identical *rrn* operons: *rrnA* through *rrnH* (*rrnF* was renamed as *rrnG*) (supporting information, Figure S1A). This sevenfold redundancy limits the introduction of resistance caused by binding site mutations, mainly due to the recessive nature of resistance mutations that are suppressed by the presence of six wild-type alleles that lead to rapid cell death (Orelle *et al.* 2013). In addition, the multiplicity of wild-type alleles will tend to revert the mutant allele due to rapid gene conversion. This extremely low-resistance frequency is from a clinical perspective a very attractive feature for an antibiotic, and as a consequence the ribosome has remained an attractive antibacterial target despite decades of macrolide and aminoglycoside usage. From a drug-discovery perspective, however, this redundancy makes definition of a structural foundation guiding iterative chemistry programs challenging.

One recent example is negamycin, a natural compound that mediates its antibacterial activity by inhibition of translation (Mizuno *et al.* 1970) and for which as many as 10 different ribosomal-binding sites have been described (Schroeder *et al.* 2007; Olivier *et al.* 2014; Polikanov *et al.* 2014a). The location of a single binding site through which the compound mediated its activity, required for structure-based design of improved analogs, remained elusive for 40 years. Negamycin-resistant mutants of wild-type *E. coli* have been isolated, but the mutations affected transport of the compound into the cell rather than binding to the ribosome (McKinney *et al.* 2015). To obtain genetic evidence for negamycin’s functional binding site, helix 34 of the small subunit, the redundancy of wild-type *E. coli* ultimately needed to be circumvented by the use of markerless single *rrn* strains (Olivier *et al.* 2014; Polikanov *et al.* 2014a). Although this tool has found use in various seminal scientific advances of ribosomal biology (Bollenbach *et al.* 2009; Orelle *et al.* 2013; Polikanov *et al.* 2014b; Orelle *et al.* 2015), its construction was mentioned in brief (Bollenbach *et al.* 2009). Here we describe the construction in detail, showing that sequential deletion of four *rrn* genes leads to a reduced growth rate due to limiting expression of transfer RNAs (tRNAs), and that after deletion of the sixth copy the cellular ribosomal content becomes growth-limiting. Furthermore, although colony morphology suggested stability of the strains (Bollenbach *et al.* 2009), whole-genome analyses revealed elevated genomic instability caused by homologous recombination between the scars remaining upon *rrn* deletion.

## Materials and Methods

### Supplementary Information

File S1 contains a list of strains (Table S1), a detailed *rrn* deletion strategy (Figure S1, Figure S2, and Figure S3) and a map of pK4-16 (Figure S5). Additional whole-genome sequencing data are provided in Figure S4.

### Data availability

*E. coli* strains SQ37, SQ88, SQ2203, SQ110, and SQ171, used for genomic sequencing, were obtained from the Coli Genetic Strain Center at Yale University. Complete nucleotide sequences of these strains have been deposited in Genbank under accession numbers CP011320, CP011321, CP011322, CP011323, and CP011324, respectively.

## Results and Discussion

A set of single *rrn* deletion strains was constructed using *E. coli* MG1655 (Blattner *et al.* 1997). Seven strains, each with a single deletion spanning one of the *rrn* operons, were made using the recombineering method (Figure S1 and Table S1) (Datsenko and Wanner 2000). Upon resolving the kanamycin-resistance cassette from one operon, leaving a 85-bp insertion (“scar”), we introduced a new kanamycin-resistance cassette into another *rrn* operon, which was subsequently resolved, and so on (Figure S2 and Figure S3A). Deletions of the *rrn* operons were confirmed by both polymerase chain reaction (data not shown) and Southern blots (Figure S3B). However, all *rrn* operons encode the RNA components for the small and large ribosomal unit (*rrs* and *rrl* genes, respectively) that are interspersed with a number of different tRNA genes that would have prevented removal of more than five operons (Figure S1B). Therefore, plasmid ptRNA67 (Zaporojets *et al.* 2003) was introduced to provide tRNA genes of Ala-1B, Ile-1, Trp, Asp-1, Thr-1, and Glu-2 *in trans* (Asai *et al.* 1999).

At high growth rates, as much as 70% of *E. coli*’s resources are devoted to the translation machinery and protein synthesis (Russell and Cook 1995). The ribosome forms the core of the translation machinery and the growth rate is proportional to both cellular ribosome content and specific peptide elongation rate (Dennis *et al.* 2004). Feedback regulation is one mechanism of ribosomal RNA regulation in *E. coli*. *E. coli* strains with a limited number of *rrn* operons maintain ribosome content by increased transcription of the remaining operons; conversely, the presence of additional *rrn* copies does not change ribosome content (Gyorfy *et al.* 2015).

**Figure 1:**
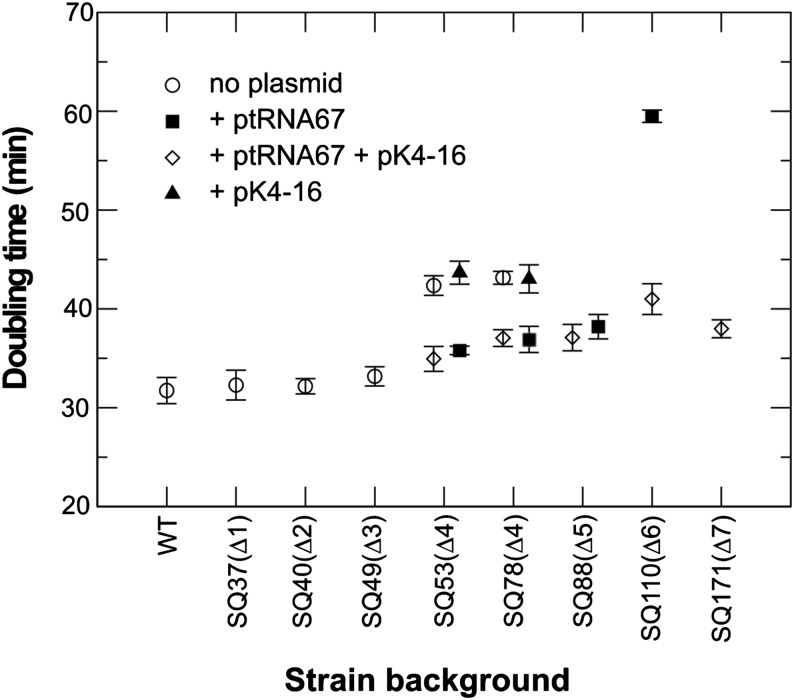
Growth rates of *E. coli rrn* deletion strains grown in Luria Broth medium at 37° with and without the tRNA plasmid, ptRNA67, and ribosomal RNA plasmid, pK4-16 (n ≥ 3).

Sequential deletion of three *rrn* operons did not significantly alter the doubling time of strains when grown in Luria Broth at 37°, but the additional deletion of a fourth operon resulted in an increase from 33 to 43 min (30%) [*rrnGBA vs. rrnGADE P* = 0.0003, *rrnGBA vs. rrnGBAD P* = 0.0018 (Figure 1)]. Deletion of the fifth operon, *rrnH*, required the addition of tRNA genes on a plasmid since this deletion would remove all copies of the tRNA-Ile and tRNA-Ala genes encoded within the *rrn* operon spacers (Figure S1B). Despite the removal of the fifth operon, the growth rate of the Δ*rrnGADEH* ptRNA67 strain was significantly higher than that of the Δ*rrnGADE* strain, with doubling time of 38 and 43 min respectively (*P* = 0.0089), suggesting that the lower growth rate of the Δ*rrnGADE* strain was caused largely by limiting amounts of tRNA (Figure 1). This observation was confirmed by failure to increase the growth rate of the Δ*rrnGADE* strain upon introduction of *rrn* encoding plasmid pK4-16 (*P* = 0.87). The largest decrease in growth rate, increasing the doubling time from 38 to 60 min, was observed upon deletion of the sixth *rrn* copy, leaving a single chromosomal *rrn* operon (Figure 1). Introduction of pK4-16 restored the growth rate close to the level observed in the Δ*rrnGADEH* ptRNA strain (*P* = 0.00002).

**Figure 2:**
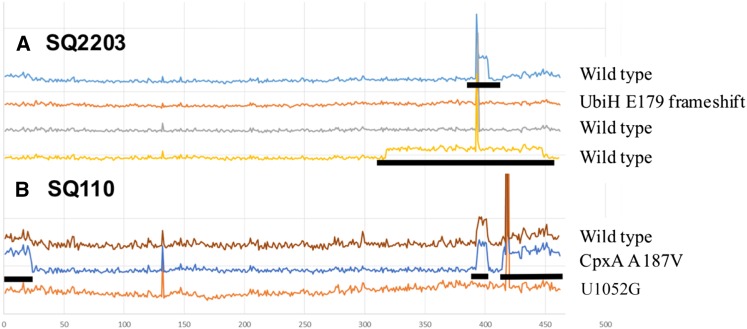
Relative sequence read coverage of multiple isolates of *E. coli* strains SQ2203 and SQ110 and negamycin-resistant mutants derived thereof (Olivier *et al.* 2014, McKinney *et al.* 2015) reveal unstable regions of genomic duplication. Regions that showed ∼2× increased relative coverage, reflecting duplication, are indicated with a black bar. (A). SQ2203 yielded two variants (blue, yellow) in additional to the predicted, single-fold coverage genome (gray). (B) All wild-type SQ110 isolates that were sequenced contained the same duplication (brown) but different arrangements were found among negamycin-resistant mutants. “Spikes” in the plots represent regions of homology of mainly transfer RNA and rrn genes/operons in resident plasmids, with genomic sequences.

For the single *rrn* strains to serve as genetic tools and select for mutations in *rrn*, whole-genome-sequences of strains SQ37, SQ88, SQ2203, SQ110, and SQ171 were determined. Mapping of the sequence reads confirmed correct deletion of the appropriate *rrn* operons, and the density of sequence reads in SQ37, containing a single deletion, and SQ88, containing five deletions, was evenly distributed across the genome (Figure S4). However, in single *rrn* operon strains SQ2203, SQ110 and SQ171 genomic regions were found in which the density of sequence reads was doubled, reflecting DNA duplications (Figure S4). The duplications always seemed to have occurred between two scar sites. In an extension of this work, whole-genome-sequences of multiple colonies of SQ110 and SQ2203 as well as negamycin-resistant mutants derived therefrom (Olivier *et al.* 2014, McKinney *et al.* 2015) were determined, and in some of these the duplication pattern was altered (Figure 2). This observation suggests that the combination of the severe growth defect observed upon removal of the penultimate *rrn* operon (Figure 1) and the presence of six identical scar sequences spread through the genome promote recombination events.

## Supplementary Material

Supporting Information
