# Supplementary material for: Markerless Escherichia coli rrn Deletion Strains for Genetic Determination of Ribosomal Binding Sites
Source: G3 (Bethesda). 2015 Oct 4;5(12):2555–7. doi: 10.1534/g3.115.022301 (PMC4683628; doi:10.1534/g3.115.022301)
Supplement: Supporting Information [file supp_5_12_2555__index.html]

Markerless Escherichia coli rrn Deletion Strains for Genetic Determination of Ribosomal Binding Sites — Supporting Information 

# Markerless *Escherichia coli rrn* Deletion Strains for Genetic Determination of Ribosomal Binding Sites

## Supporting Information for Quan *et al.*, 2015

**Files in this Data Supplement:**

- Supporting Information - Figures S1-S5, File S1, and Table S1 (PDF, 948 KB)
- Figure S1 - Extent of the deletion of each *E. coli* ribosomal RNA operon mapped with respect to Genbank version U00096.2 of the *E. coli* genomic sequence (PDF, 234 KB)
- Figure S2 - Schematic representation and sequence of FRT scar and regions flanking the *rrn deletions* (PDF, 173 KB)
- Figure S3 - Knockout strategy of *rrn* operons in *E. coli* MG1655 (PDF, 495 KB)
- Figure S4 - Relative sequence read coverage of indicated strains mapped against *E. coli* MG1655 (GenBank reference NC000913) (PDF, 199 KB)
- Figure S5 - Schematic diagram of pK4-16 (*rrnB* plasmid with pSC101 ori) (PDF, 75 KB)
- File S1 - References Supporting Information (PDF, 68 KB)
- Table S1 - Strains and plasmids used in this work (PDF, 71 KB)
