# Supplementary material for: Markerless Escherichia coli rrn Deletion Strains for Genetic Determination of Ribosomal Binding Sites
Source: G3 (Bethesda). 2015 Oct 4;5(12):2555–7. doi: 10.1534/g3.115.022301 (PMC4683628; doi:10.1534/g3.115.022301)
Supplement: Supporting Information [file supp_g3.115.022301_022301SI.pdf]

Markerless *Escherichia coli rrn* Deletion Strains for Genetic Determination of Ribosomal Binding Sites

Selwyn Quan[*], Ole Skovgaard[†], Robert E. McLaughlin[‡], Ed T. Buurman[‡,1], and Catherine L. Squires[§]

[*]Department of Biology, Stanford University, Stanford, California 94305, [†]Department of Science, Systems and Models, Roskilde University, Roskilde, Denmark DK-4000, [‡]Department of Biosciences, Infection Innovative Medicines Unit, AstraZeneca R&D Boston, Waltham, Massachusetts 02451 and [§]Department of Molecular Biology and Microbiology, Tufts University School of Medicine, Boston, Massachusetts 02111;

[1]Corresponding author: Pfizer Vaccine Research and Development, 401 North Middletown Road, Pearl River, New York 10964; E-mail: Ed.Buurman@pfizer.com

Table S1 Strains and plasmids used in this work

Strain	Genotype	Source/Reference
MG1655	*ilvG rfb-50 rph-1*	Blattner *et al*. 1997
SQ11	Δ*rrn*E::KmR	This work
SQ16	Δ*rrn*B::KmR	This work
SQ20	Δ*rrn*G::KmR	This work
SQ22	Δ*rrn*A::KmR	This work
SQ24	Δ*rrn*D::KmR	This work
SQ26	Δ*rrn*H::KmR	This work
SQ34	Δ*rrn*C::KmR	This work
SQ37	Δ*rrn*E	This work
SQ40	Δ*rrn*EG	This work
SQ49	Δ*rrn*GBA	This work
SQ53	Δ*rrn*GBAD	This work
SQ2062	Δ*rrn*GBAD(ptRNA67)	This work
SQ2066	Δ*rrn*GBAD(pK4-16)	This work
SQ2200	Δ*rrn*GBAD(pK4-16, ptRNA67)	This work
SQ78	Δ*rrn*GADE	This work
SQ2068	Δ*rrn*GADE(pK4-16)	This work
SQ2197	Δ*rrn*GADE(pK4-16, ptRNA67)	This work
SQ2199	Δ*rrn*GADE(ptRNA67)	This work
SQ88	Δ*rrn*GADEH(ptRNA67)	This work
SQ2196	Δ*rrn*GADEH(pK4-16, ptRNA67)	This work
SQ110	Δ*rrn*GADBHC(ptRNA67)	This work
SQ2194	Δ*rrn*GADBHC(pK4-16, ptRNA67)	This work
SQ141	Δ*rrn*GADEHB(pKK3535, ptRNA67)	This work
SQ2202	Δ*rrn*GADEHB(pK4-16, ptRNA67)	This work
SQ2203	Δ*rrn*GADEHB(ptRNA67)	This work
SQ171	Δ*rrn*GADEHBC(pKK3535, ptRNA67)	This work
SQ2158	Δ*rrn*GADEHBC(pK4-16, ptRNA67)	This work
Plasmid		
pKK3535	pBR322 ori, *rrn*B	Brosius *et al*. 1981
pK4-16	pSC101 ori, *rrn*B	This work
ptRNA67		Zaporojets *et al*. 2003
pKD46		Datsenko and Wanner 2000
pCP20		Cherepanov and Wackernagel 1995
pKD13		Datsenko and Wanner 2000

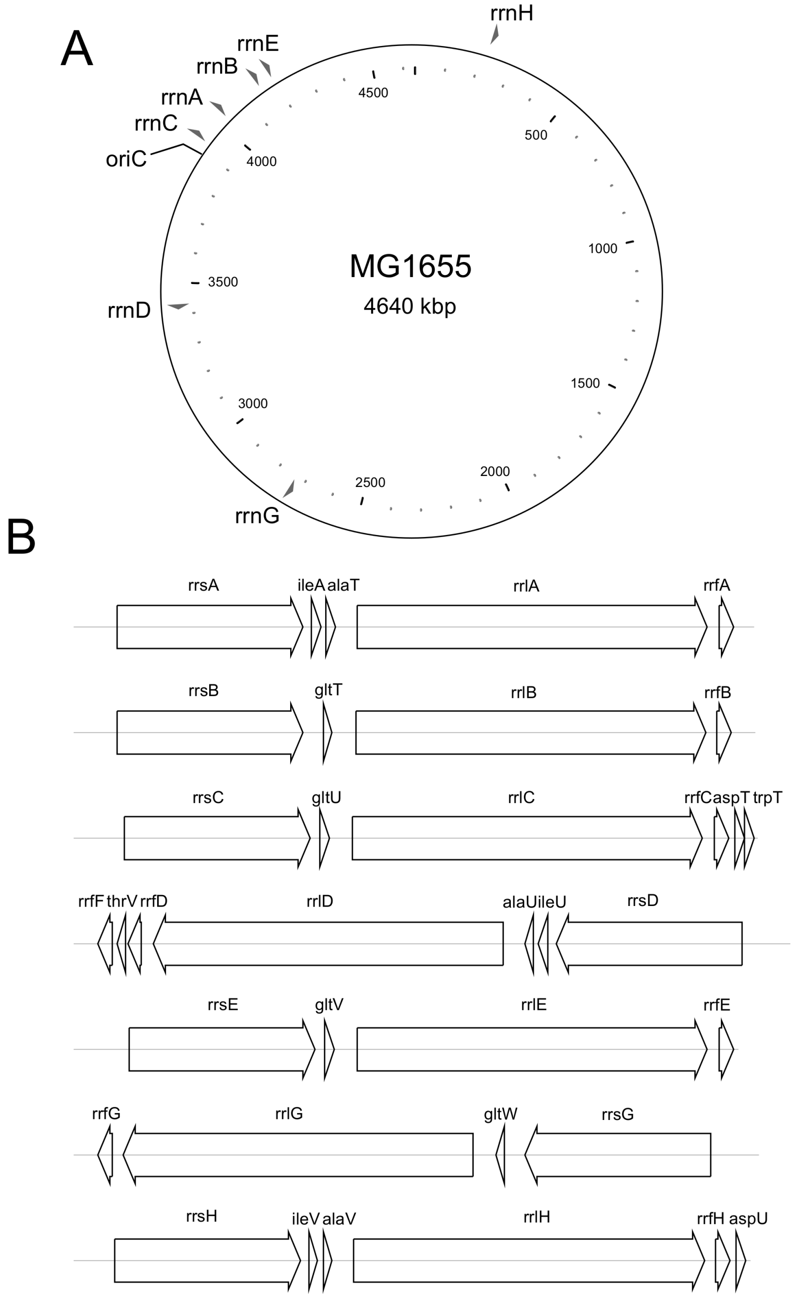

Figure S1 Extent of the deletion of each *E. coli* ribosomal RNA operon mapped with respect to Genbank version U00096.2 of the *E. coli* genomic sequence.

rrnA

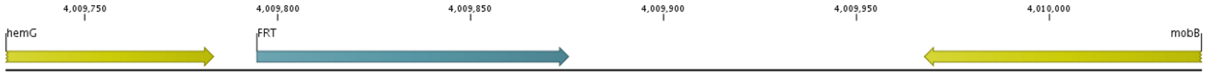

4035164..4040815
GCGAATTTCGCCCGAGAAATCGCCCATTTAACCGACAAACCGACGCTGAAATAAGCATAAAG
AATGTGTAGGCTGGAGCTGCTTCGAAGTTCCTATACTTTCTAGAGAATAGGAACTTCGAACT
GCAGGTCGACGGATCCCCGGAATAGAAGGCCATCCTGACGGATGGCCTTTTTGCATTGGCGC
AGAAAAAAATGCCGGATGCGACGCTGGCGCGTCTTATCCAGCCTACTCTTGCTTATCCGTTC
TGCTTTTGCATCCACTCCACCACAAAATCAGCCAGCCCCTCAACATCATTAATATCCAGTAA

rrnB

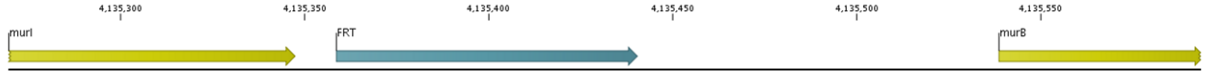

4166298..4171959
CCAGGAGCTGAACAATTATTGCCCGTTTTACAGCGTTACGGCTTCGAAACGCTCGAAAAACT
GGCAGTTTTAGGCTGATTTGGTTGAATGTGTAGGCTGGAGCTGCTTCGAAGTTCCTATACTT
TCTAGAGAATAGGAACTTCGAACTGCAGGTCGACGGATCCCCGGAATTTTTTGCGTTTCTAC
AAACTCTTCCTGTCGTCATATCTACAAGCCATCCCCCCACAGATACGGTAAACTAGCCTCGT
TTTTGCATCAGGAAAGCAGCTATGAACCACTCCTTAAAACCCTGGAACACATTTGGCATTGA
TCATAATGCTCAGC

rrnC

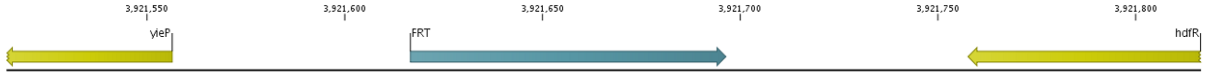

3941387..3947067
AAGGTTTTTCTGTGCAGCTAACTGTTGTGCGCTTAAAGGCATTACTTATCTTCCTTTTTCTT
TTTATTCCTCCTTAGTATGCCACCAGGAAGTGTGATTACGTGTAGGCTGGAGCTGCTTCGAA
GTTCCTATACTTTCTAGAGAATAGGAACTTCGAACTGCAGGTCGACGGATCCCCGGAATATC
TGAAATAACCCTCTCCGAAGTAAATCCTTCTACCGGCATCCTTGCCAGCCATTCATATTAAT
ACACTTCATCCAGCACGTTAATTTTCAAAAGATCGCGAATCAACGCATTTTTAT

rrnD

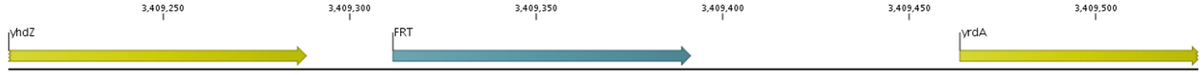

3423217..3429164
AGCAAGCTGCACCTGATGAATTTTTTGCGCATCCTAAATCAGAGCGTACGAGGGCATTTTTA
TCGCAGGTAATCCATTAATTGAATGTTAGTTCGAAAAGCAAGTGTAGGCTGGAGCTGCTTCG
AAGTTCCTATACTTTCTAGAGAATAGGAACTTCGAACTGCAGGTCGACGGATCCCCGGAATA
CTAAAAGACTTGCACAAGGCCAATAATGCCCCCAAAGTCATTAGTAAATCATTTATTGCTGA
GGTAAGTATGTCTGATGTTTTACGCCCATACCGCGATCTTTTTCCACAAATCGGTCAGCGCG
TAATGATCG

rrnE

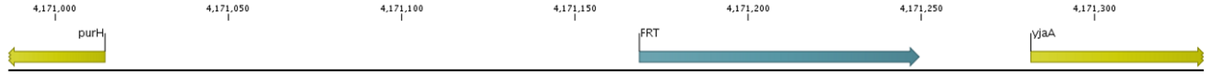

4207686..4213202
CGCGGCGGACTGGACGACGTTGTTGCATGGTAAATCCCCTGGATTTGACTATTACAGAGAGC
GTTAGCTGAATTTTTCGCGAAAAACTCAGCTAACGCCCCTAACGGGGCATCCTTATTTTTCG
CCCGCATTGTAACGAAAACGTTTGCGCAACGCTCGCGAATTTTTCTCTTTCAATGGTGGTGT
AGGCTGGAGCTGCTTCGAAGTTCCTATACTTTCTAGAGAATAGGAACTTCGGAATAGGAACT

AAGGAGGATATTCATATGTCAATTATTTGCATGATGAAGGGAATCTCATGTCAGTTCTGTAT
ATCCAAATTCGTCGTAATCAAATTACTGTTCGCGA

rrnG

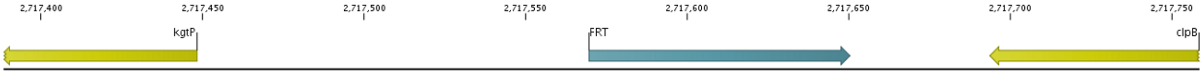

2725867..2731558
GCGGCGACGAGTATCACTACTTGTCAGTTTGCTGTCTGCCGTTACAGTACTTTCAGCCATGC
CATTATGTCTCCTGCCGTAATCCGATGCTTTTGTCGGTCGCTTTTGTTTATTTTTTTTGTAA
AGGAAATATTATACATTTGTTGCATATCATTATGCAACCTTAACCATGAATTTAGTTGTGTA
GGCTGGAGCTGCTTCGAAGTTCCTATACTTTCTAGAGAATAGGAACTTCGAACTGCAGGTCG
ACGGATCCCCGGAATACTTATAGACAAAAACGAGCCCCGAAGGGCTCGTTTTATCATTTACT
GGACGGCGACAATCCGGTCTTCATTAACTTCCAGGCGAATCACTTTACCCGGAACCAATT

rrnH

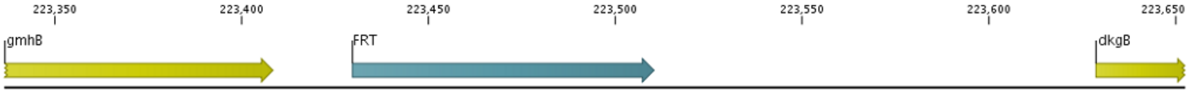

223429..229049
GCGGATTGGGTGTTAAATAGCCTGGCAGACCTGCCGCAAGCGATAAAAAAGCAGCAAAAACC
GGCACAATGATTAAAAGATGAGCGGTTGAAAGTGTAGGCTGGAGCTGCTTCGAAGTTCCTAT
ACTTTCTAGAGAATAGGAACTTCGAACTGCAGGTCGACGGATCCCCGGAATGTATATCTATT
ATTGCCAGAATCGCAAAAATCCTCTGCATTTTACGCTCTTTTTCCTCAACAGTCTGAAGCCC
ATAATCACCTCAGTTAACGAAAATAGCATTAAAAGAGGCATATTATGGCTATCCCTGCATTT
GGTTTA

Figure S2 Schematic representation and sequence of FRT scar and regions flanking the *rrn* deletions. The FRT scar sequence is denoted as underlined text. The coordinates of the deleted regions are provided above the sequence data. Coordinates are based on the sequence of *E. coli* MG1655 (NC000913: modification date 19-Mar-2014).

A

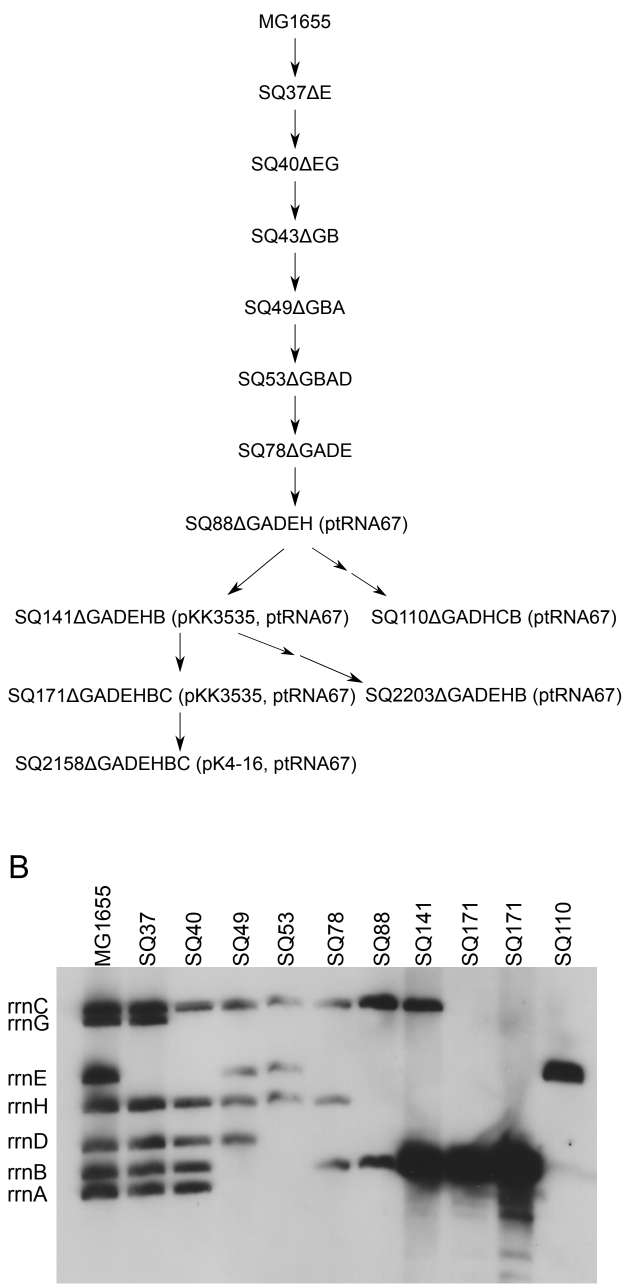

Figure S3 Knockout strategy of *rrn* operons in *E. coli* MG1655. (A) Order of ribosomal deletions to generate an *E. coli* strain with no chromosomal rRNA operons. (B) Confirmation of the *rrn* deletions by Southern blot using a 600 bp *rrs* probe.

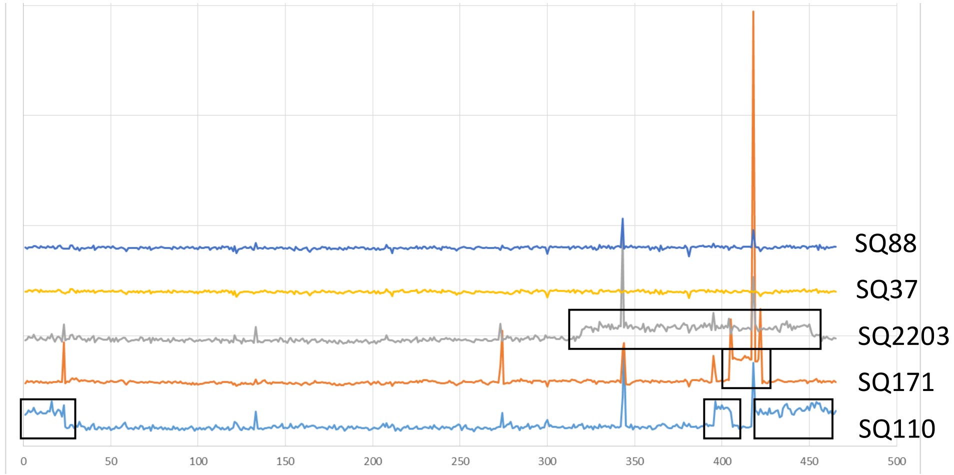

Figure S4 Relative sequence read coverage of indicated strains mapped against *E. coli* MG1655 (GenBank reference NC000913). The plot represents the average read density over 10kb windows normalized to the average genome coverage. Regions of duplication are represented by ~2x relative coverage and indicated as boxed areas on the plot. 'Spikes' in the plots represent resident plasmids, containing regions with homology to the genomic DNA, incl. tRNA and rRNA regions.

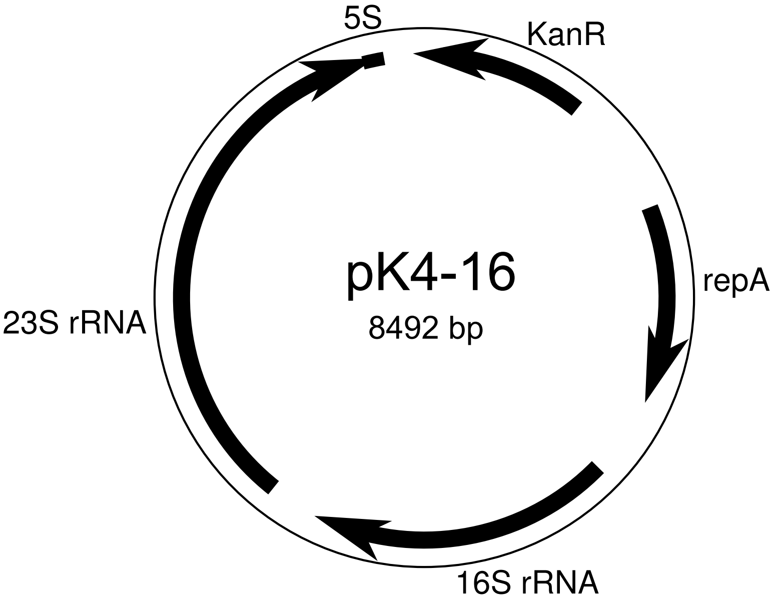

Figure S5 Schematic diagram of pK4-16 (*rrn*B plasmid with pSC101 ori)

File S1
References Supporting Information

Blattner FR, Plunkett G, Bloch CA, Perna NT, V. Burland *et al*., 1997 The complete genomic sequence of *Escherichia coli* K-12. Science 277: 1453-1474.

Brosius, J., A. Ullrich, M. A. Raker, A. Gray, T. J. Dull *et al*., 1981 Construction and fine mapping of recombinant plasmids containing the *rrnB* ribosomal RNA operon of *E. coli*. Plasmid 6: 112-118.

Cherepanov, P. P., and W. Wackernagel, 1995 Gene disruption in *Escherichia coli*: TcR and KmR cassettes with the option of Flp-catalyzed excision of the antibiotic-resistance determinant. Gene 158: 9–14

Datsenko, K. A., and B. L. Wanner, 2000 One-step inactivation of chromosomal genes in *Escherichia coli* K-12 using PCR products. Proc. Natl. Acad. Sci. USA 97: 6640-6645.

Zaporojets, D., S. French, and C. L. Squires, 2003 Products transcribed from rearranged *rrn* genes of *Escherichia coli* can assemble to form functional ribosomes. J. Bacteriol. 185: 6921-6927.
