# Supplementary material for: Markerless Escherichia coli rrn Deletion Strains for Genetic Determination of Ribosomal Binding Sites
Source: G3 (Bethesda). 2015 Oct 4;5(12):2555–7. doi: 10.1534/g3.115.022301 (PMC4683628; doi:10.1534/g3.115.022301)
Supplement: Supporting Information [file supp_g3.115.022301_FigureS1.pdf]

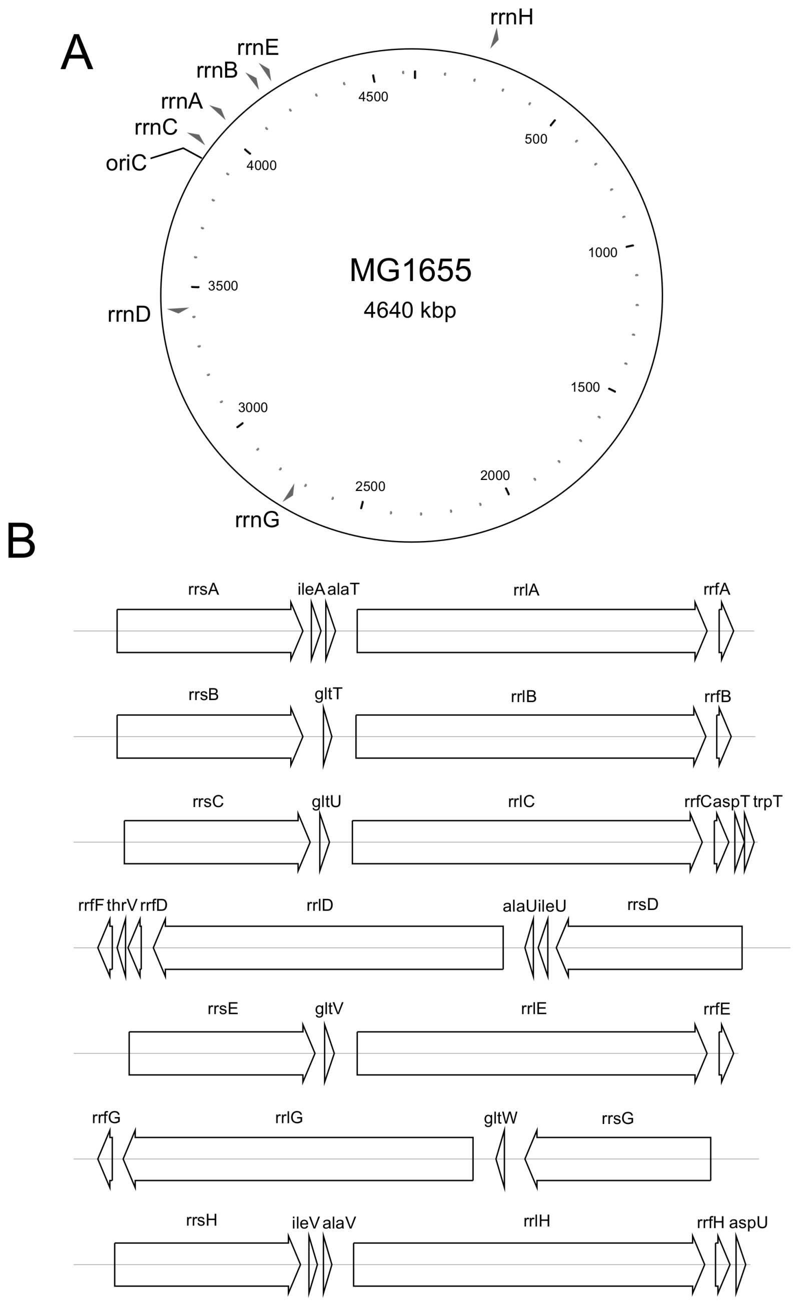

Figure S1 Extent of the deletion of each *E. coli* ribosomal RNA operon mapped with respect to Genbank version U00096.2 of the *E. coli* genomic sequence.
