# Supplementary material for: Markerless Escherichia coli rrn Deletion Strains for Genetic Determination of Ribosomal Binding Sites
Source: G3 (Bethesda). 2015 Oct 4;5(12):2555–7. doi: 10.1534/g3.115.022301 (PMC4683628; doi:10.1534/g3.115.022301)
Supplement: Supporting Information [file supp_g3.115.022301_FigureS2.pdf]

rrnA

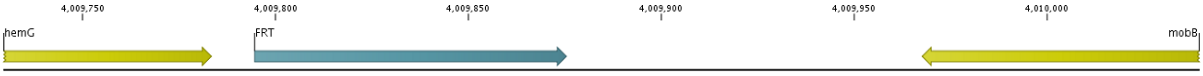

4035164..4040815
GCGAATTTCGCCCGAGAAATCGCCCATTTAACCGACAAACCGACGCTGAAATAAGCATAAAG
AATGTGTAGGCTGGAGCTGCTTCGAAGTTCCTATACTTTCTAGAGAATAGGAACTTCGAACT
GCAGGTCGACGGATCCCCGGAATAGAAGGCCATCCTGACGGATGGCCTTTTTGCATTGGCGC
AGAAAAAAATGCCGGATGCGACGCTGGCGCGTCTTATCCAGCCTACTCTTGCTTATCCGTTC
TGCTTTTGCATCCACTCCACCACAAAATCAGCCAGCCCCTCAACATCATTAATATCCAGTAA

rrnB

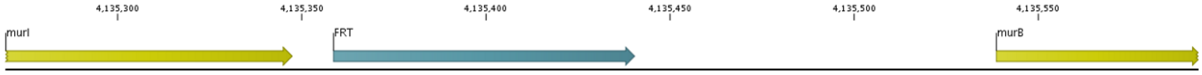

4166298..4171959
CCAGGAGCTGAACAATTATTGCCCGTTTTACAGCGTTACGGCTTCGAAACGCTCGAAAAACT
GGCAGTTTTAGGCTGATTTGGTTGAATGTGTAGGCTGGAGCTGCTTCGAAGTTCCTATACTT
TCTAGAGAATAGGAACTTCGAACTGCAGGTCGACGGATCCCCGGAATTTTTTGCGTTTCTAC
AAACTCTTCCTGTCGTCATATCTACAAGCCATCCCCCCACAGATACGGTAAACTAGCCTCGT
TTTTGCATCAGGAAAGCAGCTATGAACCACTCCTTAAAACCCTGGAACACATTTGGCATTGA
TCATAATGCTCAGC

rrnC

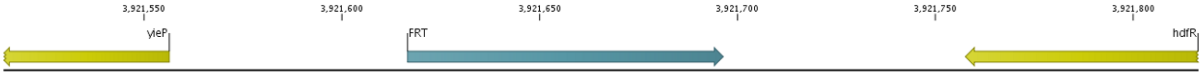

3941387..3947067
AAGGTTTTTCTGTGCAGCTAACTGTTGTGCGCTTAAAGGCATTACTTATCTTCCTTTTTCTT
TTTATTCCTCCTTAGTATGCCACCAGGAAGTGTGATTACGTGTAGGCTGGAGCTGCTTCGAA
GTTCCTATACTTTCTAGAGAATAGGAACTTCGAACTGCAGGTCGACGGATCCCCGGAATATC
TGAAATAACCCTCTCCGAAGTAAATCCTTCTACCGGCATCCTTGCCAGCCATTCATATTAAT
ACACTTCATCCAGCACGTTAATTTTCAAAAGATCGCGAATCAACGCATTTTTAT

rrnD

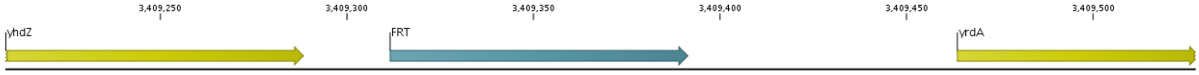

3423217..3429164
AGCAAGCTGCACCTGATGAATTTTTTGCGCATCCTAAATCAGAGCGTACGAGGGCATTTTTA
TCGCAGGTAATCCATTAATTGAATGTTAGTTCGAAAAGCAAGTGTAGGCTGGAGCTGCTTCG
AAGTTCCTATACTTTCTAGAGAATAGGAACTTCGAACTGCAGGTCGACGGATCCCCGGAATA
CTAAAAGACTTGCACAAGGCCAATAATGCCCCCAAAGTCATTAGTAAATCATTTATTGCTGA
GGTAAGTATGTCTGATGTTTTACGCCCATACCGCGATCTTTTTCCACAAATCGGTCAGCGCG
TAATGATCG

rrnE

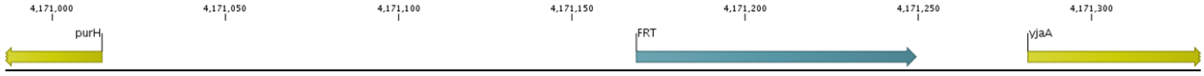

4207686..4213202
CGCGGCGGACTGGACGACGTTGTTGCATGGTAAATCCCCTGGATTTGACTATTACAGAGAGC
GTTAGCTGAATTTTTCGCGAAAAACTCAGCTAACGCCCCTAACGGGGCATCCTTATTTTTCG
CCCGCATTGTAACGAAAACGTTTGCGCAACGCTCGCGAATTTTTCTCTTTCAATGGTGGTGT
AGGCTGGAGCTGCTTCGAAGTTCCTATACTTTCTAGAGAATAGGAACTTCGGAATAGGAACT

AAGGAGGATATTCATATGTCAATTATTTGCATGATGAAGGGAATCTCATGTCAGTTCTGTAT
ATCCAAATTCGTCGTAATCAAATTACTGTTCGCGA

rrnG

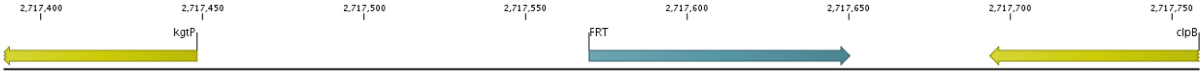

2725867..2731558
GCGGCGACGAGTATCACTACTTGTCAGTTTGCTGTCTGCCGTTACAGTACTTTCAGCCATGC
CATTATGTCTCCTGCCGTAATCCGATGCTTTTGTCGGTCGCTTTTGTTTATTTTTTTTGTAA
AGGAAATATTATACATTTGTTGCATATCATTATGCAACCTTAACCATGAATTTAGTTGTGTA
GGCTGGAGCTGCTTCGAAGTTCCTATACTTTCTAGAGAATAGGAACTTCGAACTGCAGGTCG
ACGGATCCCCGGAATACTTATAGACAAAAACGAGCCCCGAAGGGCTCGTTTTATCATTTACT
GGACGGCGACAATCCGGTCTTCATTAACTTCCAGGCGAATCACTTTACCCGGAACCAATT

rrnH

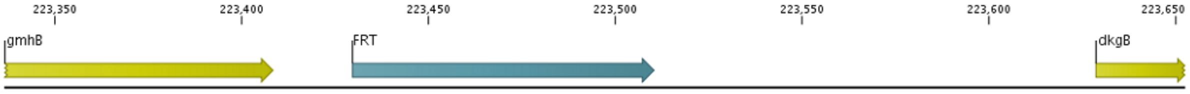

223429..229049
GCGGATTGGGTGTTAAATAGCCTGGCAGACCTGCCGCAAGCGATAAAAAAGCAGCAAAAACC
GGCACAATGATTAAAAGATGAGCGGTTGAAAGTGTAGGCTGGAGCTGCTTCGAAGTTCCTAT
ACTTTCTAGAGAATAGGAACTTCGAACTGCAGGTCGACGGATCCCCGGAATGTATATCTATT
ATTGCCAGAATCGCAAAAATCCTCTGCATTTTACGCTCTTTTTCCTCAACAGTCTGAAGCCC
ATAATCACCTCAGTTAACGAAAATAGCATTAAAAGAGGCATATTATGGCTATCCCTGCATTT
GGTTTA

Figure S2 Schematic representation and sequence of FRT scar and regions flanking the *rrn* deletions. The FRT scar sequence is denoted as underlined text. The coordinates of the deleted regions are provided above the sequence data. Coordinates are based on the sequence of *E. coli* MG1655 (NC000913: modification date 19-Mar-2014).
