# Supplementary material for: Markerless Escherichia coli rrn Deletion Strains for Genetic Determination of Ribosomal Binding Sites
Source: G3 (Bethesda). 2015 Oct 4;5(12):2555–7. doi: 10.1534/g3.115.022301 (PMC4683628; doi:10.1534/g3.115.022301)
Supplement: Supporting Information [file supp_g3.115.022301_FigureS3.pdf]

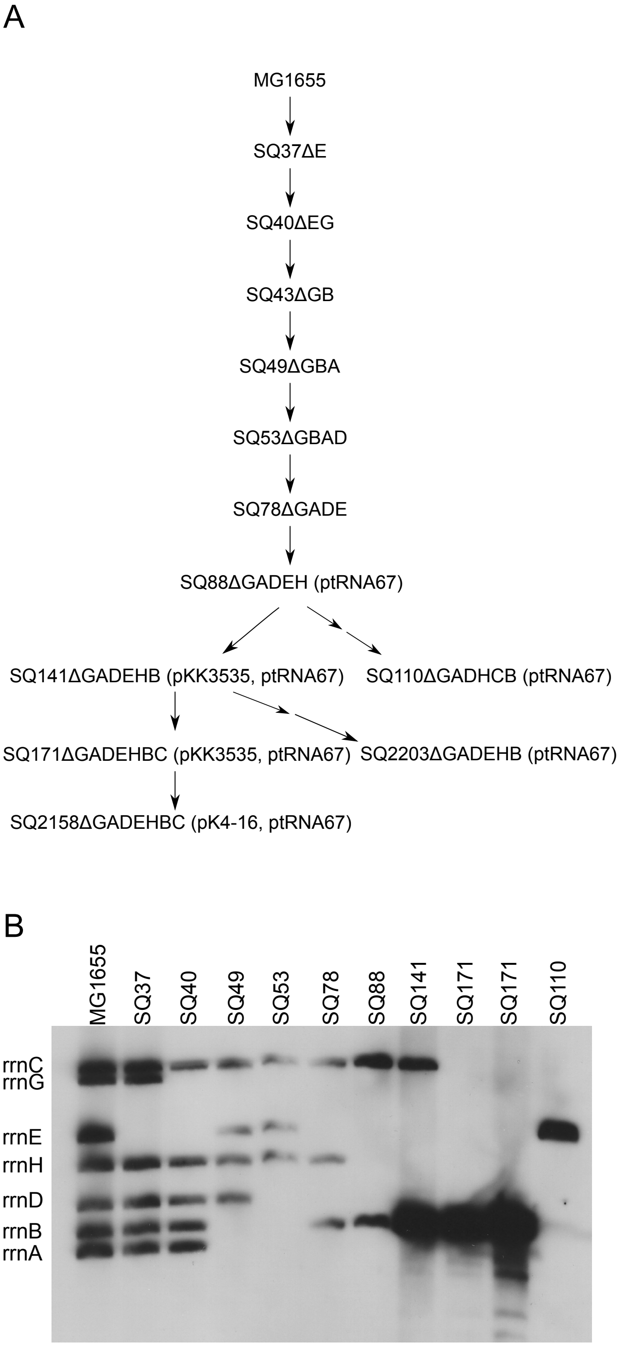

Figure S3 Knockout strategy of *rrn* operons in *E. coli* MG1655. (A) Order of ribosomal deletions to generate an *E. coli* strain with no chromosomal rRNA operons. (B) Confirmation of the *rrn* deletions by Southern blot using a 600 bp *rrs* probe.
