# Supplementary material for: Markerless Escherichia coli rrn Deletion Strains for Genetic Determination of Ribosomal Binding Sites
Source: G3 (Bethesda). 2015 Oct 4;5(12):2555–7. doi: 10.1534/g3.115.022301 (PMC4683628; doi:10.1534/g3.115.022301)
Supplement: Supporting Information [file supp_g3.115.022301_FigureS4.pdf]

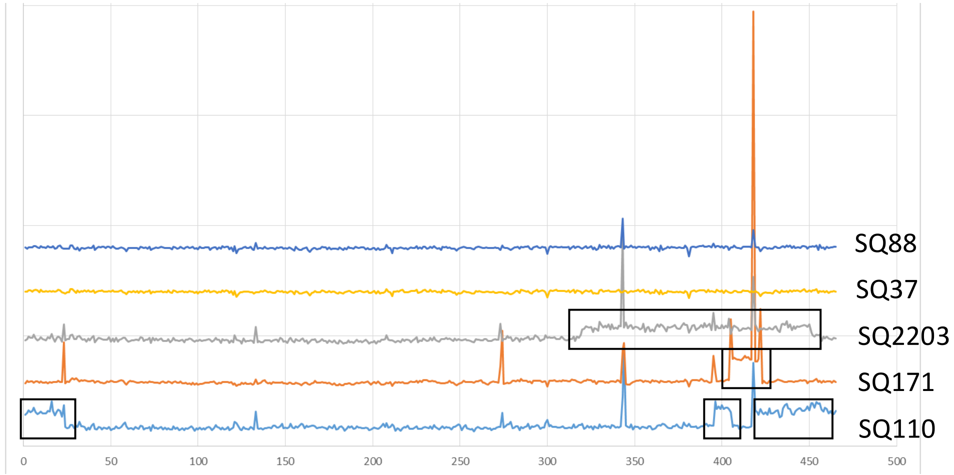

Figure S4 Relative sequence read coverage of indicated strains mapped against *E. coli* MG1655 (GenBank reference NC000913). The plot represents the average read density over 10kb windows normalized to the average genome coverage. Regions of duplication are represented by ~2x relative coverage and indicated as boxed areas on the plot. 'Spikes' in the plots represent resident plasmids, containing regions with homology to the genomic DNA, incl. tRNA and rRNA regions.
