# Supplementary figures and images for: Markerless Escherichia coli rrn Deletion Strains for Genetic Determination of Ribosomal Binding Sites
Source: G3 (Bethesda). 2015 Oct 4;5(12):2555–7. doi: 10.1534/g3.115.022301 (PMC4683628; doi:10.1534/g3.115.022301)

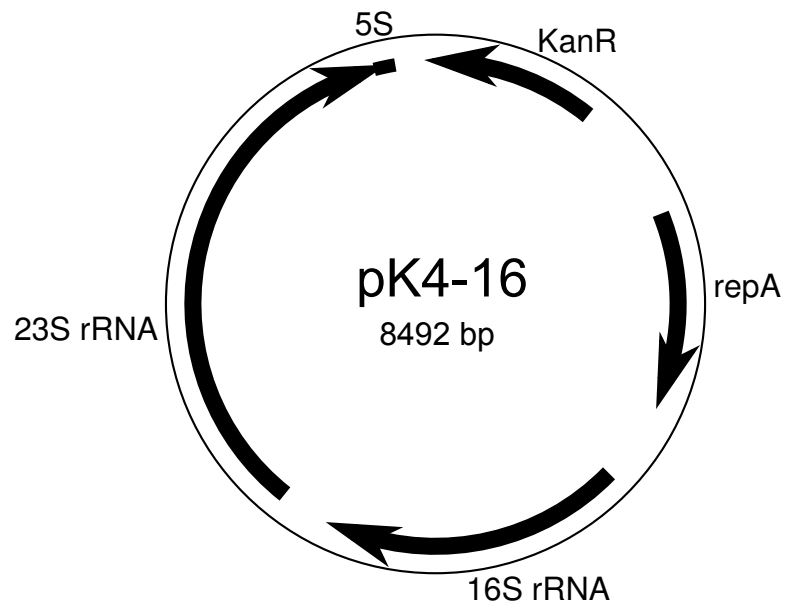

**Figure S5** Schematic diagram of pK4-16 (*rrnB* plasmid with pSC101 ori)

Supplement: Supporting Information [file supp_g3.115.022301_FigureS5.pdf]
