# Supplementary material for: Markerless Escherichia coli rrn Deletion Strains for Genetic Determination of Ribosomal Binding Sites
Source: G3 (Bethesda). 2015 Oct 4;5(12):2555–7. doi: 10.1534/g3.115.022301 (PMC4683628; doi:10.1534/g3.115.022301)
Supplement: Supporting Information [file supp_g3.115.022301_FileS1.pdf]

File S1
References Supporting Information

Blattner FR, Plunkett G, Bloch CA, Perna NT, V. Burland *et al*., 1997 The complete genomic sequence of *Escherichia coli* K-12. Science 277: 1453-1474.

Brosius, J., A. Ullrich, M. A. Raker, A. Gray, T. J. Dull *et al*., 1981 Construction and fine mapping of recombinant plasmids containing the *rrnB* ribosomal RNA operon of *E. coli*. Plasmid 6: 112-118.

Cherepanov, P. P., and W. Wackernagel, 1995 Gene disruption in *Escherichia coli*: TcR and KmR cassettes with the option of Flp-catalyzed excision of the antibiotic-resistance determinant. Gene 158: 9–14

Datsenko, K. A., and B. L. Wanner, 2000 One-step inactivation of chromosomal genes in *Escherichia coli* K-12 using PCR products. Proc. Natl. Acad. Sci. USA 97: 6640-6645.

Zaporojets, D., S. French, and C. L. Squires, 2003 Products transcribed from rearranged *rrn* genes of *Escherichia coli* can assemble to form functional ribosomes. J. Bacteriol. 185: 6921-6927.
