# Supplementary material for: Markerless Escherichia coli rrn Deletion Strains for Genetic Determination of Ribosomal Binding Sites
Source: G3 (Bethesda). 2015 Oct 4;5(12):2555–7. doi: 10.1534/g3.115.022301 (PMC4683628; doi:10.1534/g3.115.022301)
Supplement: Supporting Information [file supp_g3.115.022301_TableS1.pdf]

Table S1 Strains and plasmids used in this work

Strain	Genotype	Source/Reference
MG1655	*ilvG rfb-50 rph-1*	Blattner *et al*. 1997
SQ11	Δ*rrn*E::KmR	This work
SQ16	Δ*rrn*B::KmR	This work
SQ20	Δ*rrn*G::KmR	This work
SQ22	Δ*rrn*A::KmR	This work
SQ24	Δ*rrn*D::KmR	This work
SQ26	Δ*rrn*H::KmR	This work
SQ34	Δ*rrn*C::KmR	This work
SQ37	Δ*rrn*E	This work
SQ40	Δ*rrn*EG	This work
SQ49	Δ*rrn*GBA	This work
SQ53	Δ*rrn*GBAD	This work
SQ2062	Δ*rrn*GBAD(ptRNA67)	This work
SQ2066	Δ*rrn*GBAD(pK4-16)	This work
SQ2200	Δ*rrn*GBAD(pK4-16, ptRNA67)	This work
SQ78	Δ*rrn*GADE	This work
SQ2068	Δ*rrn*GADE(pK4-16)	This work
SQ2197	Δ*rrn*GADE(pK4-16, ptRNA67)	This work
SQ2199	Δ*rrn*GADE(ptRNA67)	This work
SQ88	Δ*rrn*GADEH(ptRNA67)	This work
SQ2196	Δ*rrn*GADEH(pK4-16, ptRNA67)	This work
SQ110	Δ*rrn*GADBHC(ptRNA67)	This work
SQ2194	Δ*rrn*GADBHC(pK4-16, ptRNA67)	This work
SQ141	Δ*rrn*GADEHB(pKK3535, ptRNA67)	This work
SQ2202	Δ*rrn*GADEHB(pK4-16, ptRNA67)	This work
SQ2203	Δ*rrn*GADEHB(ptRNA67)	This work
SQ171	Δ*rrn*GADEHBC(pKK3535, ptRNA67)	This work
SQ2158	Δ*rrn*GADEHBC(pK4-16, ptRNA67)	This work
Plasmid		
pKK3535	pBR322 ori, *rrn*B	Brosius *et al*. 1981
pK4-16	pSC101 ori, *rrn*B	This work
ptRNA67		Zaporojets *et al*. 2003
pKD46		Datsenko and Wanner 2000
pCP20		Cherepanov and Wackernagel 1995
pKD13		Datsenko and Wanner 2000
